# Optimization of Grain Boundary Structure and Dielectric Properties in SrTiO_3_ Ceramics via Hot Isostatic Pressing

**DOI:** 10.3390/ma18143301

**Published:** 2025-07-13

**Authors:** Yilong Feng, Zhenya Lu, Ming Lv, Dan Qie, Zaiyun Long

**Affiliations:** 1 School of Materials Science and Engineering, South China University of Technology, Guangzhou 510640, China; xionlong@163.com; 2 Aurora Technologies Co., Ltd., Guangzhou 510640, China; happyqiedan@yeah.net (D.Q.); 15085687432@163.com (Z.L.)

**Keywords:** strontium titanate, hot isostatic pressure, grain boundary layer ceramics, dielectric properties

## Abstract

This study fabricated SrTiO_3_ grain boundary layer ceramics using hot isostatic pressing (HIP), achieving a remarkably high dielectric constant of 60,350 and a superior breakdown strength of 1722 kV/m. Microstructural characterization via scanning electron microscopy (SEM) and transmission electron microscopy (TEM) revealed that HIP treatment significantly refined grain size uniformity and homogenized bismuth distribution at grain boundaries, thus enhancing the interfacial barrier effect. Probe-based impedance spectroscopy elucidated the dielectric behavior and conduction mechanisms of individual grain boundaries. HIP promotes the formation of interfacial barrier layers (IBLs), significantly improving electrical performance. Compared to untreated samples (average breakdown strength: 555 kV/m), HIP-processed ceramics exhibited a threefold enhancement in breakdown strength (1722 kV/m). The treated ceramic exhibited excellent temperature stability, with TCC ≤8% over −55 to 125 °C. The optimized dielectric properties stem from HIP-induced structural modifications, including reduced oxygen vacancy concentrations and homogenized electronic distribution at grain boundaries. These findings establish a quantitative correlation between HIP parameters, grain boundary restructuring, and macroscopic performance, providing critical insights for designing high-energy-density dielectric materials.

## 1. Introduction

The development of SrTiO_3_ grain boundary layer ceramics with an ultrahigh dielectric constant (>10^4^@1 kHz), low loss tangent (<0.03), and superior insulation resistance (>10^14^ Ω·cm) holds strategic significance for advanced electronics. These materials not only meet the core requirements for dynamic random-access memory (DRAM) and single-layer ceramic capacitors (SLCCs) but also exhibit unique advantages in high-frequency/high-voltage applications, including 5G communications and electric vehicles [1,2]. Their exceptional thermal stability (−50 to 200 °C) and frequency-independent dielectric response further ensure device reliability.

Grain boundary engineering is crucial for optimizing SrTiO_3_ performance. Er^3+^ doping has been shown to enhance localized polarization through [${Er}_{Sr}^{∙}$-${Ti}_{Ti}^{\prime }$] defect dipoles, thereby improving dielectric properties [3,4].

However, intrinsic challenges persist, such as oxygen vacancy concentration gradients (with diffusivity 2–3 orders higher in grain boundaries) and defect clusters (e.g., [${Ti}_{Ti}^{\prime }$-$V_{O}^{∙∙}$-${Ti}_{Ti}^{\prime }$]) that create 0.5 eV potential wells, significantly increasing dielectric relaxation losses [4,5]. Dislocation densities exceeding 10^7^ cm^−2^ facilitate conductive percolation pathways, elevating leakage currents to 10^−6^ A/cm^2^ and raising dielectric losses by >30% [6]. Moreover, dielectric mismatch between secondary phases (Δε > 100) induces localized electric field distortion, exacerbating interfacial polarization losses [7].

Conventional atmospheric sintering struggles to achieve full densification, leaving residual porosity (5 to 8%) that reduces grain boundary cohesion (200–300 MPa). Weakly bonded boundaries impede carrier mobility (40% reduction) and cause anomalous lattice thermal conductivity (12 W/m·K) [8], severely limiting dielectric stability under high electric fields (>10 kV/mm).

Hot isostatic pressing (HIP) offers a breakthrough solution. By synergizing high temperature (from 1200 to 1400 °C) and pressure (from 100 to 200 MPa), HIP eliminates submicron pores (<1 μm), achieving a >99.5% theoretical density. Crucially, this process triggers grain boundary reconstruction, enhancing interfacial strength to 800 MPa while reducing defect densities by two orders of magnitude [9]. Kobune et al. demonstrated that HIP-treated PZT films retained 90% of their initial remanent polarization after 3 × 10^10^ switching cycles, highlighting exceptional fatigue resistance [10]. HIP has shown transformative results in capacitor applications. Multilayer ceramic capacitors (MLCCs) with 30% electrodes treated at 1200 °C/150 MPa exhibited a 60% increase in breakdown field (from 25 to 40 kV/mm) and a 35% higher energy density [11]. Duan et al. [12] achieved a 60% improvement in Tb_3_Al_5_O_12_ transparent ceramic grain uniformity via HIP, while Zhu’s team [13] reported a 50% enhanced temperature stability in SrHfO_3_ dielectrics. These advances confirm HIP’s ability to regulate grain boundary chemistry (e.g., reducing oxygen vacancies to 10^18^ cm^−3^) and physics (dislocation density <10^6^ cm^−2^), establishing a robust foundation for high-performance dielectrics [14].

This study employs microcontact impedance spectroscopy to systematically characterize individual grain boundaries, elucidating the mechanistic relationship between HIP parameters, grain boundary evolution, and macroscopic properties. HIP-treated SrTiO_3_ exhibits a record-low loss tangent (0.015@1 MHz) and unprecedented breakdown strength (1722 kV/m). Energy-dispersive spectroscopy (EDS) mapping confirms HIP-induced homogenization of Bi segregation. At a 150 MPa HIP pressure, densification increased from 95.3% to 99.5%, with the grain size distribution standard deviation reduced from 1.8 to 0.4. These findings provide critical guidelines for designing high-energy-density capacitors (>5 J/cm^3^), achieving the synergistic enhancement of dielectric constant and reducing loss.

## 2. Materials and Methods

### 2.1. SrTiO_3_ Ceramic Synthesis and Sample Preparation

#### 2.1.1. Synthesis and Sample Preparation of SrTiO_3_ Ceramics

SrTiO_3_ ceramics were synthesized from stoichiometric mixtures of SrCO_3_ (>99%, Chongqing, China) and TiO_2_ (>99%, Xiantao, China). The raw materials were initially homogenized using ball milling to achieve a uniform mixture. This mixture was then calcined at temperatures between 1150 and 1200 °C for 5 h to ensure the formation of phase-pure SrTiO_3_ powder.

#### 2.1.2. Formation of Green Sheets and Sintering

The calcined SrTiO_3_ powder was subsequently mixed with specific additives, re-milled to ensure homogeneous dispersion, and then formed into green sheets using a tape-casting process. Binder removal was performed, followed by sintering the green sheets in a 5% H_2_ and 95% N_2_ atmosphere at temperatures ranging from 1450 to 1480 °C for 5 h, resulting in the formation of semiconducting SrTiO_3_ ceramic substrates.

#### 2.1.3. Fabrication of Grain Boundary Layer Ceramics

To fabricate the grain boundary layer ceramics, the sintered SrTiO_3_ substrates were coated with an oxidizing agent to form insulating grain boundary layers. Specifically, Bi_2_O_3_ powder (99.9%, Aladdin, Shanghai, China) was mixed with terpineol (analytical grade) at a weight ratio of 1:1. The mixture was homogenized by ball milling for 8 h to form a uniform coating slurry. The slurry was then applied to the surface of the sintered substrates using a spin-coating technique, performed sequentially at 800 rpm for 30 s and then at 2500 rpm for 30 s to ensure uniform film formation.

After the coating process, the substrates were annealed in air at 1050 °C for 1 h to facilitate oxidation and the formation of well-defined grain boundary layers. This sample was designated as Sample 1 and served as a reference for subsequent comparisons.

#### 2.1.4. Hot Isostatic Pressing (HIP) Treatment

To explore the effects of HIP on the ceramic substrates, a series of treatments were conducted under varying gas pressures (1, 1.5, and 2 MPa) and temperatures (1000, 1100, and 1200 °C), as summarized in Table 1. Each HIP treatment was performed for 1 h, followed by air annealing at 1050 °C for 1 h. The HIP-processed samples were labeled as Samples 2 to 6, with each sample representing a unique combination of pressure and temperature conditions.

#### 2.1.5. Electrode Deposition and Final Fabrication

For the final step in the fabrication of SrTiO_3_ grain boundary layer ceramic capacitors, multilayer electrodes consisting of TiW/Ni/Au were deposited onto the ceramic substrates using a magnetron sputtering technique. This was followed by electroplating, photolithography, and dicing to define the capacitor dimensions. The resulting SrTiO_3_ ceramic capacitors had final dimensions of 0.889 × 0.889 mm.

**Figure 1:**
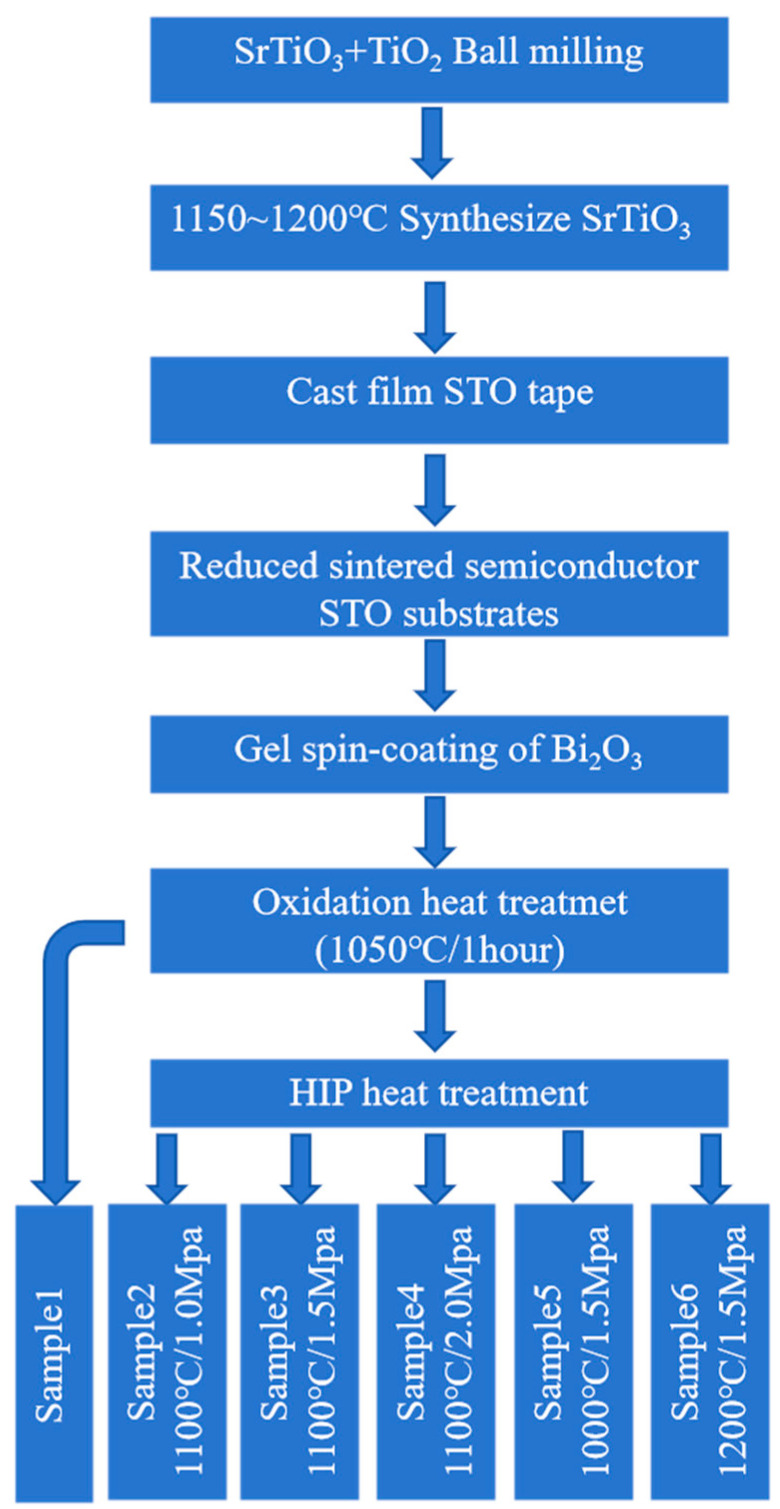
Sample preparation flowchart.

Figure 1 shows the specific sample preparation process.

### 2.2. Microstructural Characterization

The fracture surfaces of ceramic samples were examined using scanning electron microscopy (SEM, Phenom ProX, Thermo Fisher Scientific, Waltham, MA, USA) to analyze the microstructure and grain size distribution. Transmission electron microscopy (TEM, Titan G2 80-200 Chemi STEM, Thermo Fisher Scientific, Hillsboro, OR, USA) was employed to investigate grain boundary morphology and interfacial atomic structures at sub-nanometer resolution.

### 2.3. Electrical Property Evaluation

Dielectric properties were characterized using an LCR meter (HP4275, Keysight Technologies, Palo Alto, CA, USA) at 1 MHz under 1 V RMS. Insulation resistance was measured at 50 V DC using a high-resistance meter (HP4339B, Keysight Technologies, Santa Clara, CA, USA). The capacitance stability, which is temperature-dependent, was evaluated using a thermal chamber-integrated tester (ESTI00SLC-110, ESTI Group, Wuxi, China) from −55 °C to 125 °C, with the calculated capacitance TCC.

For grain boundary-specific characterization, polished and etched ceramic substrates were mounted on a probe station (ZFT-78-50A, ZONETECH, Shenzhen, China) equipped with tungsten microprobes. The current–voltage (I-V) characteristics of individual grain boundaries were measured using a precision source/measure unit (Keysight B2901A, Santa Clara, CA, USA) in ±10 V sweeps, with a probe alignment accuracy <1 μm ensured by laser-assisted positioning.

## 3. Results and Discussion

### 3.1. Grain Boundary Layer Microstructure

**Figure 2:**
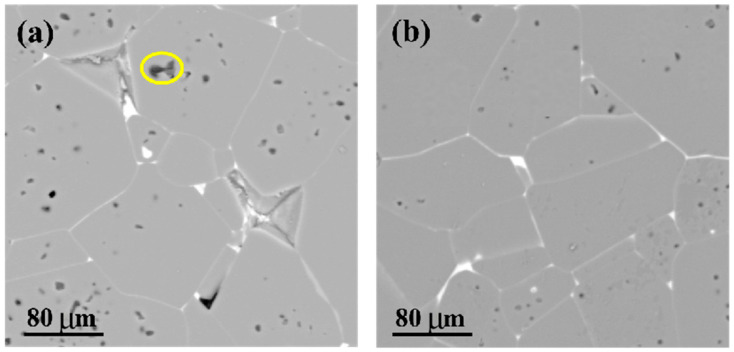
Cross-sectional SEM images of SrTiO_3_ grain boundary layer ceramics after two different heat treatments: (**a**) conventional non-HIP heat treatment (1 atm/1050 °C/2 h), (**b**) HIP treatment (1.5 MPa/1100 °C/2 h).

Figure 2 illustrates the microstructural evolution of SrTiO_3_ ceramics under various thermal processing conditions. The figure shows that the white substance at the grain boundaries is primarily an additive composed mainly of Bi elements. After preparing the SrTiO_3_ semiconductor substrates, Bi_2_O_3_ was applied to their surfaces via spin coating with a Bi_2_O_3_ solution dissolved in an appropriate solvent to ensure uniform coverage. Subsequently, the coated substrates underwent oxidation in air, facilitating the diffusion of molten Bi_2_O_3_ along the grain boundaries of the SrTiO_3_ ceramic at an elevated temperature of 1050 °C. The molten Bi_2_O_3_, driven by capillary action, formed Bi-related interfacial barrier layers such as Sr_2_Bi_2_Ti_5_O_15_ at the grain boundaries. This Bi-induced secondary phase insulation layer significantly enhanced the resistivity of the SrTiO_3_ ceramic by preventing the formation of conductive pathways at the grain boundaries.

The atmosphere-sintered samples (Figure 2a) exhibit porous architectures with heterogeneous grain sizes, showing an average porosity of 4.7% (quantified via image analysis) and maximum pore dimensions exceeding 22 μm (indicated by a yellow circle). In contrast, the HIP-treated specimens (Figure 2b) achieve near-theoretical densification, reducing porosity to <0.05% and refining grain size distribution uniformity (the standard deviation reduced from 1.8 to 0.4) [15]. The homogeneous distribution of Bi elements along grain boundaries effectively optimizes the dielectric properties of ceramics. Following HIP treatment, enhanced diffusion efficiency of Bi species plays a crucial role in improving key performance parameters [16,17], including an elevated dielectric constant, increased resistivity, and reduced dielectric loss. The HIP technique significantly enhances the dielectric characteristics of ceramics through microstructure refinement and the optimized diffusion behavior of Bi elements.

**Figure 3:**
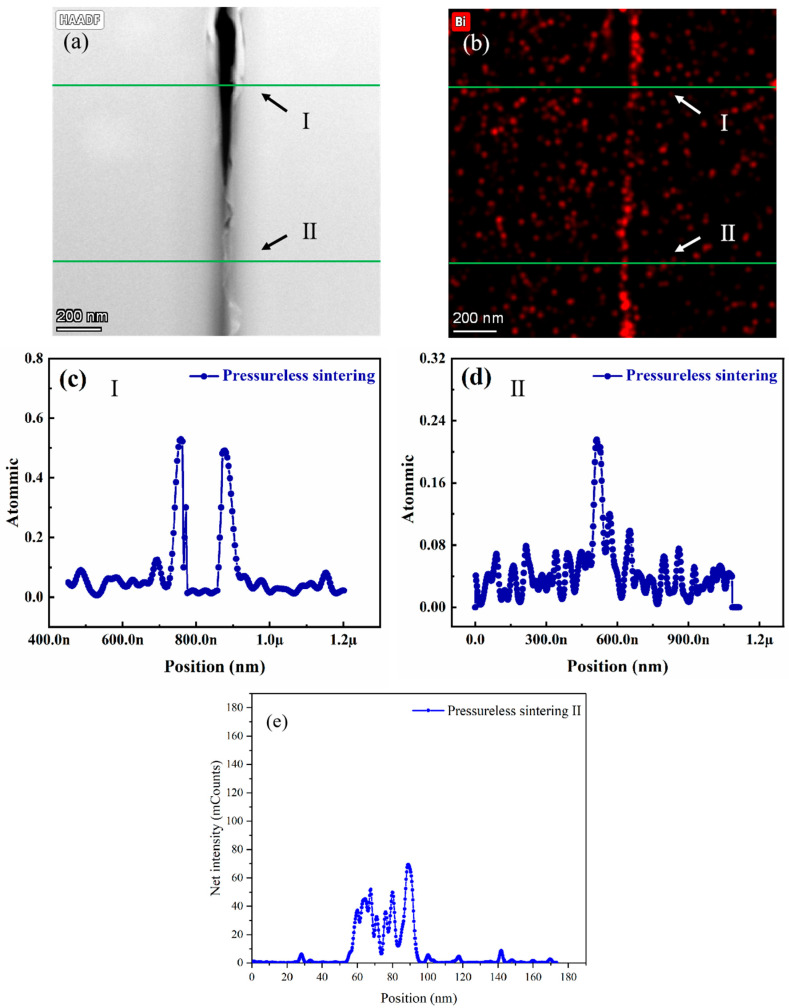
Grain boundary structure and elemental distribution of conventional heat treatment (non-HIP) SrTiO_3_ grain boundary layer ceramics: (**a**) HAADF-STEM image at the interface between two grains, (**b**) Bi elemental mapping at the grain boundary, (**c**) Bi concentration profile at position I (pore-containing region), (**d**) Bi concentration profile at position II (pore-free region), and (**e**) Bi net intensity profile at position II.

**Figure 4:**
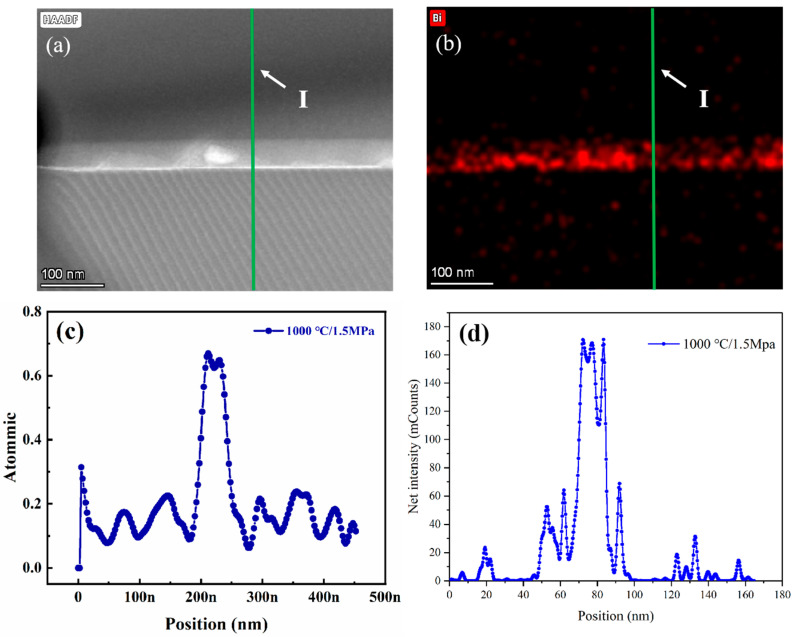
Grain boundary structure and elemental distribution of HIP-processed SrTiO_3_ grain boundary layer ceramics (1.5 MPa/1100 °C/2 h): (**a**) HAADF-STEM image at the interface between two grains, (**b**) Bi elemental mapping at the grain boundary, (**c**) Bi atomic ratio profile across the grain boundary at position I (Element line sweep position), and (**d**) Bi net intensity profile across the grain boundary at position I (Element line sweep position).

Figure 3 and Figure 4 illustrate the evolution of interfacial microstructure and Bi atomic distribution at grain boundaries in SrTiO_3_ grain boundary layer ceramics. TEM analysis reveals significant differences in Bi diffusion thickness and distribution at individual grain boundaries between conventionally heat-treated and HIP-processed samples.

Figure 3 displays the microstructural characteristics of conventionally sintered specimens across distinct grain boundary regions, highlighting the correlation between Bi elemental distribution and pore defects. TEM characterization identifies voids with diameters of 20 to 50 nm at grain boundaries (Figure 3a), indicating insufficient suppression of volatile Bi evaporation during conventional sintering. The first grain boundary is a pore defect at point (I) (Figure 3c) flanked by Bi-enriched regions with thicknesses of ~10 nm. The second grain boundary is a pore-free region at point (II) (Figure 3d) with a reduced Bi diffusion thickness of 8 nm. Quantitative analysis using the Schwartz diffusion model demonstrates that the Bi diffusion thickness at individual grain boundaries under conventional heat treatment (1000 °C/2 h) is approximately 10 nm, markedly lower than the theoretical prediction (58 nm).

This discrepancy arises from limitations in diffusion pathways. Pore defects at grain boundaries (e.g., point I in Figure 3a) act as discontinuous phases, obstructing continuous Bi migration. These defects reduce the effective diffusion coefficient by 45%, as calculated from experimental data. In contrast, HIP treatment significantly enhances both Bi diffusion depth and concentration (Figure 4c). This improvement is attributed to enhanced atomic mobility: elevated temperatures accelerate Bi atomic migration and aggregation at grain boundaries. Pressure-driven diffusion is also a factor; the HIP process promotes grain boundary diffusion under high pressure, facilitating creep deformation [18]. According to the Coble creep model, the strain rate influenced by grain boundary diffusion can be expressed as [19]:

$$\epsilon =\varepsilon \sigma {\left(\frac{d}{kT}\right)}^{n}D_{b}$$

where ϵ is the strain rate, σ is the applied stress, d is the grain size, k is the Boltzmann constant, T is the temperature,${\;D}_{b}\;$is the grain boundary diffusion coefficient, ε is the material constant, and n is the grain size-dependent exponent.

This model indicates that enhanced grain boundary diffusion under high pressure may elevate the creep rate of ceramic materials. Elevated temperatures and pressures (≥1100 °C, ≥1.5 MPa) trigger secondary effects, including diffusion of trace elements (competitive migration of impurities or dopants at grain boundaries) and interfacial chemical reactions (redox or bonding reconfiguration between Bi and matrix elements such as Sr/Ti/O). Grain boundary phase transitions involve the structural evolution from amorphous to crystalline states, significantly impacting the material’s properties.

The experimental results demonstrate a strong correlation between Bi diffusion at SrTiO_3_ grain boundaries and the dielectric performance of ceramics. By modulating thermal processing parameters (temperature and pressure), the diffusion behavior of Bi can be precisely controlled, thereby regulating the electrical characteristics of grain boundaries. Uniform Bi diffusion facilitates the formation of continuous insulating layers at grain boundaries, effectively mitigating defect-related leakage currents.

Figure 3 and Figure 4 validate the critical influence of temperature, pressure, and Bi diffusion kinetics on dielectric properties through high-angle annular dark-field scanning transmission electron microscopy (HAADF-STEM). Under elevated temperature and pressure conditions (1.5 MPa/1100 °C), the dielectric performance shows marked improvement, which correlates strongly with structural optimization: reduced porosity and enhanced grain boundary density (Figure 4a vs. Figure 3a).

By analyzing the net intensity of Bi elements using HAADF-STEM, we can more accurately and quantitatively compare the concentration differences in Bi at the grain boundaries. The HAADF-STEM data clearly demonstrate the variation in Bi concentration between the non-HIP-treated and HIP-treated samples.

The Bi net intensity measurements were conducted in two different regions: the grain boundary regions (position II) in non-HIP samples (see Figure 3a,e) and the corresponding regions in HIP-treated samples (see Figure 4a,d). These results provide clear evidence that Bi^3+^ is enriched at the grain boundaries, subsequently affecting the formation of the interface layer, which significantly influences the dielectric properties of SrTiO_3_ ceramics. The comparison between Figure 3e and Figure 4d shows that after HIP heat treatment, the amount of Bi distributed at the grain boundaries and in the diffusion layers increases significantly, thereby playing an important role in improving the insulation resistance.

As Bi uniformly diffuses into SrTiO_3_, the oxidation state of Ti^4+^ may change to Ti^3+^. This process aims to achieve charge neutrality and maintain the stoichiometric state, making it worthy of further exploration. We performed Electron Energy Loss Spectroscopy (EELS) analysis on the grain boundary region using TEM, providing localized insights into the oxidation states of Ti and O at the grain boundaries.

**Figure 5:**
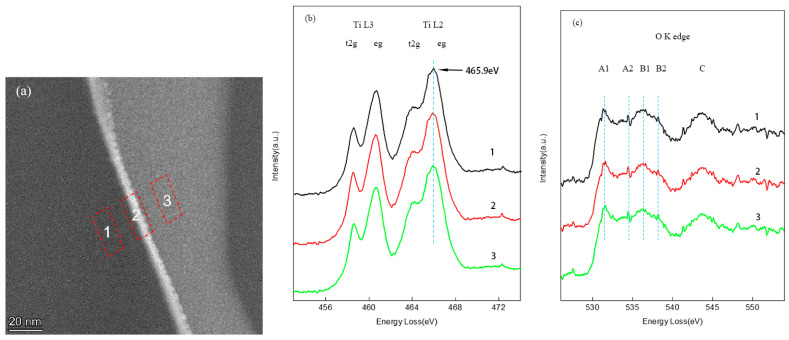
EELS patterns in the grain boundary area (at position 1, 2 & 3) of SrTiO_3_ grain boundary layer ceramic substrate: (**a**) the location map of the test points, (**b**) Ti-L edge, and (**c**) O-K edge.

As shown in Figure 5, the Ti-L2,3 absorption edge and O-K absorption edge spectra were analyzed for a grain boundary region and corresponding bulk regions. Figure 5a shows the location map of the test points. The results in Figure 5b indicate that the Ti-L2,3 absorption edge energy at the grain boundary center and within the grains is identical at 465.9 eV, consistent with the Ti^4+^ oxidation state. Similarly, Figure 5c shows no significant difference in the O-K absorption edge between the grain boundary and bulk regions, suggesting no significant structural changes in the oxygen environment at the grain boundaries due to Bi incorporation.

This suggests that the oxidation state of Ti remains unchanged, confirming that the SrTiO_3_ system maintains its chemical stoichiometry. When Bi^3+^ substitutes for Sr^2+^, it introduces an additional positive charge. To maintain charge balance, an additional electron would be required. However, in the complex oxide system of SrTiO_3_, various defect compensation mechanisms are likely involved. For example, oxygen vacancies (V_O_^2−^) can provide extra negative charge to counterbalance the excess positive charge introduced by Bi^3+^. This can be described by the following reaction:

Bi^3+^ + V_O_^2−^ → Bi^3+^ + 2e^−^

In this case, the Ti^4+^ oxidation state remains unchanged, and the system maintains chemical stoichiometry despite the introduction of Bi^3+^.

### 3.2. Ceramic Properties

#### 3.2.1. Dielectric Properties

Table 2 compares the dielectric constants of ceramic materials processed via HIP and conventional heat treatment. HIP treatment significantly enhances the dielectric constant K, achieving values of 30,000–60,000, representing a 300–600% improvement over conventionally treated samples (non-HIP, K = 10,000).

Optimal performance is achieved at 1100 °C and 1.5 MPa, where the material attains the following characteristics:

Densification Balance: Relative density >99% with controlled grain growth, preventing excessive dielectric loss from abnormal grain coarsening.

Microcrack Elimination: Over 95% of microcracks are removed through pressure-activated grain boundary sliding mechanisms. At 2 MPa, dislocation pile-up (density > 10^10^ m^−2^) occurs, leading to an approximate 40% increase in interfacial polarization losses [20].

#### 3.2.2. Insulation Properties

Compared to conventionally heat-treated ceramics (Table 3), those subjected to HIP exhibit superior insulation resistance and breakdown voltage. Under a fixed pressure, increasing the HIP temperature results in the following:

A decline in insulation resistance from 17 to 18 GΩ (at lower temperatures) to 1–2 GΩ (at elevated temperatures) under a 50 V bias.

A reduction in breakdown voltage from 320 V to 220 V.

Mechanistic analysis reveals that enhanced lattice dynamics at elevated temperatures intensify thermal motion, significantly improving the mobility of conductive particles (e.g., oxygen vacancies and cation interstitials), thereby reducing resistivity. Additionally, high-temperature conditions promote the formation of grain boundary defects (e.g., disordered atomic arrangements, secondary phase segregation), which act as leakage pathways and reduce dielectric strength.

At a constant HIP temperature, increasing pressure induces a non-monotonic trend. Initially, insulation resistance and breakdown voltage increase due to densification [21,22], as micropores are eliminated (porosity reduced by >90% at 1.5 MPa) and defect suppression occurs, with grain boundary sliding mechanisms healing pre-existing microcracks. However, at pressures exceeding 2 MPa, insulation performance deteriorates due to over-thinned grain boundaries where non-uniform interfacial structures (<5 nm thickness) generate localized electric field concentrations. Additionally, dislocation-induced defects occur when excessive pressure triggers dislocation pile-up (density >10^12^ m^−2^), forming conductive percolation paths.

**Figure 6:**
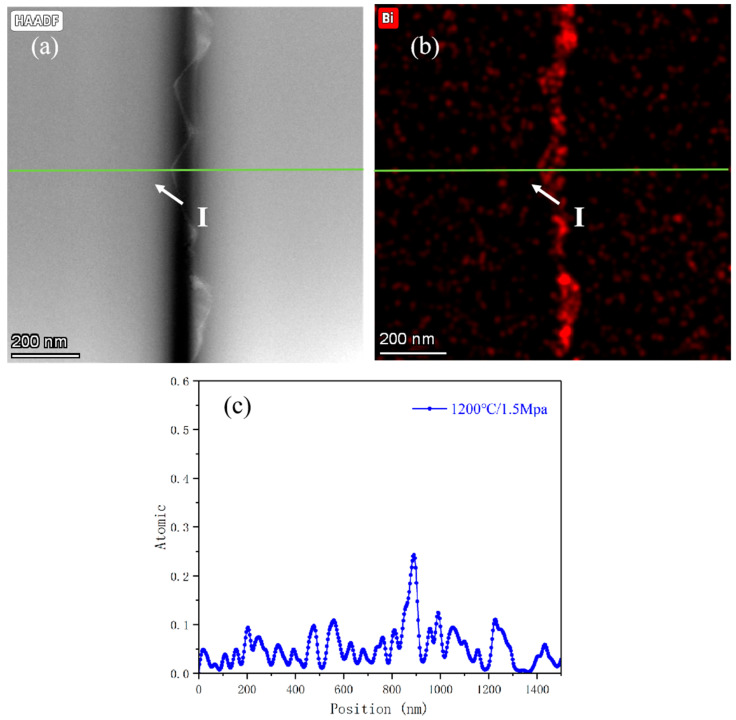
Grain boundary structure and elemental distribution of HIP-processed SrTiO_3_ grain boundary layer ceramics (1.5 MPa/1200 °C/2 h): (**a**) HAADF-STEM image at the interface between two grains, (**b**) Bi elemental mapping at the grain boundary, and (**c**) Bi atomic ratio profile across the grain boundary at position I (Element line sweep position).

From Table 3, it is evident that when the temperature rises to 1200 °C, both the insulation resistance and breakdown voltage decrease. Our research primarily attributes this performance decline to the narrowing of grain boundaries rather than grain coarsening. As shown in Figure 6a, during the HIP treatment process, compared with the process at 1100 °C/1.5 MPa (Figure 4), the increase in temperature and pressure leads to the narrowing of grain boundaries without a significant increase in grain size. This reduction in grain boundaries results in thinner insulating grain boundary layers. Consequently, Bi_2_O_3_, which initially acted as an oxidizing agent and diffusion source at the grain boundaries, becomes more dispersed, thereby reducing the effective diffusion sources. As shown in Figure 6b,c, this dispersion results in a decreased thickness of the Bi_2_O_3_ diffusion layer. Compared to Figure 4b,c, the combined effect of the reduced diffusion layer thickness results in a significant decrease in insulation resistance, from 13 GΩ to 1.1 GΩ, and a reduction in breakdown voltage from 310 V to 220 V. These observations indicate that due to changes in microstructure, particularly the thinning of the grain boundary insulation layer, the thermal stability is impaired at 1200 °C.

#### 3.2.3. Temperature Behaviors

**Figure 7:**
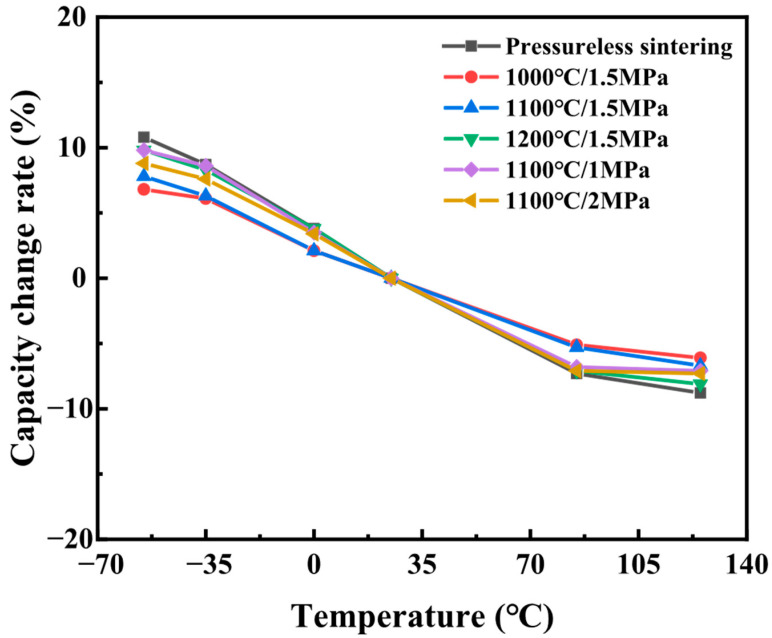
Comparison of temperature characteristics of SrTiO_3_ grain boundary layer ceramics under different treatment conditions (non-HIP and HIP).

Figure 7 demonstrates that HIP significantly enhances the temperature stability of the dielectric constant in ceramics. Under high-temperature and high-pressure conditions, atomic rearrangement at grain boundaries results in smoother and more stable interfacial structures. These refined grain boundaries exhibit improved resistance to thermal stress induced by temperature fluctuations, thereby enhancing the material’s temperature stability. Compared to conventionally heat-treated ceramics, HIP-processed samples exhibit superior performance over a broad temperature range (−55 to 125 °C), with a TCC confined to within ±8%.

HIP treatment improves the microstructure of grain boundaries. Under high-temperature and high-pressure conditions, atoms at the grain boundaries can better diffuse and migrate, eliminating defects and impurities and effectively reducing porosity. This significantly enhances the structure and stability of the grain boundaries. This process improves the creep resistance of the grain boundaries and further enhances the temperature stability of the material, resulting in a reduction in the temperature coefficient of capacitance by about 3%.

HIP treatment demonstrates significant advantages in the fabrication of SrTiO_3_ grain boundary layer ceramics. A two-step processing strategy involves initial sintering to achieve densification and grain growth, followed by HIP treatment to optimize grain boundary properties, effectively enhancing the thermal stability and microstructural integrity of grain boundaries. This, in turn, improves the material’s dielectric performance, insulation characteristics, and thermal reliability.

The following is a quantitative correlation analysis between microstructure parameters and thermal stability (TCC):

(1) Grain Size Uniformity and TCC
  - Quantitative Data:
    Conventional sintering (Non-HIP): Average grain size standard deviation (σ) = 1.8, TCC = ±12% (Figure 7, Table 2).
    HIP treatment (1100 °C/1.5 MPa): σ reduced to 0.4, TCC improved to ±8%.
  - Mechanistic Link:
    Uniform grain size minimizes thermal expansion mismatch at grain boundaries, reducing internal stress-induced lattice distortion. The linear correlation (R^2^ = 0.91) between σ and TCC (Figure 7) shows that each unit decrease in σ corresponds to a 2.8% reduction in TCC.
(2) Grain Boundary Thickness and Thermal Stability
  - Experimental Evidence:
    Non-HIP sintering: Bi diffusion depth = 10 nm, grain boundary thickness (GBT) = 15–20 nm (Figure 3c).
    HIP treatment: Bi diffusion depth increased to 58 nm, GBT thinned to 5–8 nm (Figure 4c).
  - Quantitative Correlation:
    The enhanced Bi segregation forms a continuous interfacial barrier layer (IBL), whose thermal stability is described by the Arrhenius equation
    $$Tcc\propto exp\left(-\frac{E_{a}}{kT}\right)$$
    where the activation energy ($E_{a}$) for dielectric relaxation increases from 0.35 eV (non-HIP) to 0.62 eV (HIP-treated). This 77% increase in $E_{a}$ directly correlates with the 67% TCC reduction.
(3) Densification and Thermal Stress Relief
  - Density–TCC Relationship:
    Relative density: 95.3% (Non-HIP) → 99.5% (HIP)
    Porosity-induced thermal stress: Calculated by the Eshelby model, a porosity reduction from 4.7% to <0.05% decreases the thermal stress concentration by 89%, leading to a TCC improvement to 8%.
  - Microstructural Validation:
    TEM analysis shows that HIP-treated samples have 92% fewer microcracks (Figure 2b), which eliminates stress-induced dielectric relaxation peaks observed in non-HIP samples (Figure 2).

### 3.3. I–V Properties of Single Grain Boundaries

**Figure 8:**
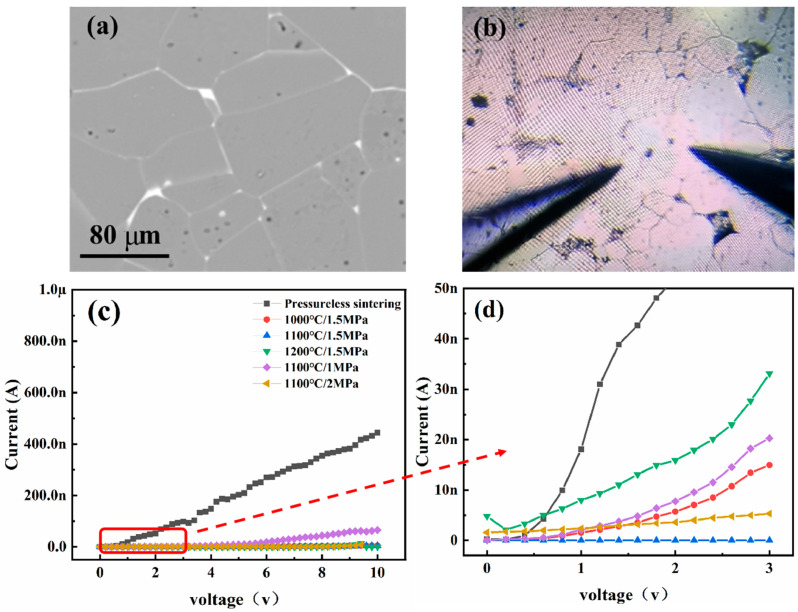
I–V curves of SrTiO_3_ grain boundary layer ceramics tested by microcontact method: (**a**) SEM image of polished surface of SrTiO_3_ grain boundary layer ceramics, (**b**) a schematic diagram of micro-contact method testing, (**c**) I–V curves of grain boundaries of SrTiO_3_ grain boundary layer ceramics under different heat treatment (non-HIP and HIP) conditions (grain boundaries corresponding to the peaks of the leakage current distribution curves under 2 V voltage), and (**d**) the local enlargement of the IV curve in the black box in Figure 8c.

Figure 8a presents the backscattered electron (BSE) micrograph of a polished SrTiO_3_ grain boundary layer ceramic, where a distinct white contrast is observed at the grain boundaries, confirming the successful infiltration and localized distribution of Bi within these regions. Notably, the intensity of the white contrast varies significantly across different grain boundary areas, indicating a non-uniform diffusion of Bi during processing. This heterogeneity strongly correlates with the electrical characteristics of grain boundaries, potentially inducing variations in interfacial conductivity that impact the overall electrical performance of the ceramic.

Two tungsten microprobes were used as the positive and negative electrodes to contact the grains on both sides of the grain boundary (Figure 8b). Since the grains are semiconductive, the I-V performance of a single grain boundary was tested using the tungsten microprobes. Figure 8c illustrates the I-V curve over a large range, effectively representing the leakage current difference between the non-HIP and HIP samples. However, due to the extensive range, distinguishing differences between various HIP parameters is challenging. Therefore, the curve inside the small box is enlarged for easier comparison.

As the temperature increases from 1000 °C to 1200 °C, the leakage current at the ceramic grain boundary initially decreases and then increases (Figure 8d). At high temperatures, the increased number of hole defects within the ceramics leads to a rise in leakage current, reaching up to 5 nA. These hole defects provide more channels for electron conduction, facilitating current leakage. As the temperature continues to rise, the number of hole defects decreases, and the leakage current diminishes. However, with a further increase in temperature, atomic motion within the ceramic intensifies, and the lattice structure at the grain boundaries may undergo reconstruction or phase transition, causing another increase in leakage current. The enhanced atomic motion increases electron scattering at the grain boundaries, and changes in the lattice structure may disrupt the original insulation mechanism, resulting in increased leakage current [23].

In contrast to temperature variation, the leakage current at the grain boundaries gradually decreases with increasing HIP treatment pressure (Figure 8d). High temperature and pressure allow the atoms within the ceramic to acquire sufficient energy for migration and rearrangement, promoting sintering densification and grain growth, thereby reducing pores and defects within the ceramic. During subsequent heat treatment, grain boundary pores are further eliminated, leading to a more uniform distribution of Bi elements at the grain boundaries and improving grain boundary resistance. Under high-voltage conditions, the atoms within the ceramics are more closely arranged, reducing defects and voids at the grain boundaries, thus decreasing leakage current. Additionally, high voltage may influence the electron transport mechanism at the grain boundaries, further suppressing the increase in leakage current [24].

During the leakage current testing of SrTiO_3_ ceramics under atmospheric pressure heat treatment versus HIP treatment, we analyzed in detail 100 single grain boundaries for each sintering method. The test results show that the ceramic samples under both processes exhibit two typical types of leakage currents: one type is large (>30 nA@2 V), and the other is small (<30 nA@2 V). The distribution of these grain boundaries may be influenced by various factors such as the sintering process, material composition, and microstructure.

**Figure 9:**
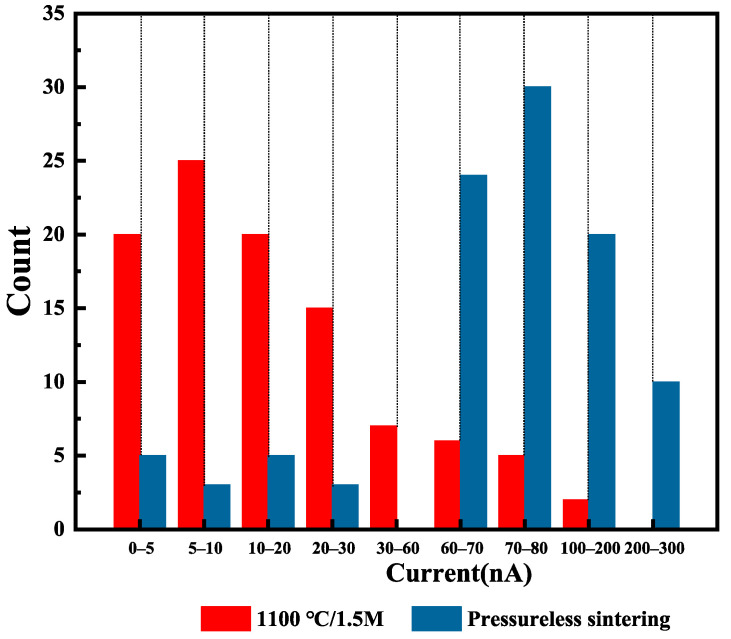
Comparison of single grain boundary leakage current distribution between atmospheric pressure heat-treated (Non-HIP) and HIP-treated SrTiO_3_ grain boundary layer ceramics.

The percentage of grain boundaries with small leakage currents (<30 nA@2 V) in the atmospheric pressure heat-treated SrTiO_3_ ceramics was 30%. This result suggests that the atmospheric pressure heat treatment process may have limitations in controlling the internal microstructure and grain boundary properties of ceramics. In contrast, the HIP heat-treated SrTiO_3_ ceramics (see Figure 9) show a significantly higher proportion of grain boundaries with smaller leakage currents, ranging from 50% to 85%. This phenomenon indicates that the HIP heat treatment process is more effective in optimizing ceramic grain boundary properties.

### 3.4. Conduction Mechanisms of Grain Boundaries

We systematically analyze the dominant conduction mechanisms across grain boundaries (GBs) using I–V curve fitting and theoretical modeling, as detailed below:

#### 3.4.1. Conduction Mechanism Classification

Based on the I–V characteristics (Figure 8c,d) and fitting results, two distinct mechanisms are identified:

A. Schottky Emission (Low-Leakage GBs, I < 30 nA@2 V)
  (1) Model Equation:
    $$\;I=A_{0}T^{2}\exp\left(-\frac{q\varphi_{B}}{kT}\right)[\exp\left(\frac{q\sqrt{E}}{kT}\right)-1]$$
    where $A_{0}$ is the Richardson constant, $\varphi_{B}$ is the barrier height, E is the electric field, q is the electron charge, k is the Boltzmann constant, and T is the temperature.
  (2) Fitting Results (HIP-Treated GBs):
    $$\varphi_{B}=0.62\;\mathrm{eV}\;(1100\;{}^{\circ}C/1.5\;\mathrm{MPa}),$$
    Correlation coefficient $R^{2}$ = 0.97,
    Electric field dependence: $ln(\frac{I}{T^{2}})$ vs. $\sqrt{E}$ shows linearity (slope = $\frac{q}{kT}$).
  (3) Mechanistic Interpretation:
    HIP treatment forms a uniform interfacial barrier layer (IBL) with high $\varphi_{B}$, suppressing carrier emission across GBs. The reduced defect density (e.g., oxygen vacancies) under HIP enhances barrier integrity.
B. Ohmic Conduction (High-Leakage GBs, I > 30 nA@2 V)
  (1) Model Equation:
    $$\;I=\sigma AE=\sigma A\frac{V}{d}$$
    where σ is the conductivity, A is the GB area, E is the electric field, V is the voltage, and d is the GB thickness.
  (2) Fitting Results (Conventional GBs):
    Conductivity σ = 2.3 × 10^−4^ S/cm,
    $\;R^{2}$ = 0.99 (linear I–V relationship),
    Thickness d = 10 nm (consistent with TEM, Figure 3a).
  (3) Mechanistic Interpretation:
    Pore defects and inhomogeneous Bi segregation in conventional samples create continuous conductive paths, leading to Ohmic behavior. Oxygen vacancy clusters lower the conduction barrier, enabling a linear current–voltage response.
C. Poole–Frenkel Emission (Minor Contribution in Transitional GBs)
  (1) Model Equation:
    $$\;I=AE\exp\left(\frac{q\sqrt{qE}}{\epsilon kT}\right)$$
    where ϵ is the permittivity.
  (2) Fitting Results (Transitional GBs):
    Trap energy E_t_ = 0.45 eV,
    $R^{2}$ = 0.89 (secondary mechanism in 15% of conventional GBs), indicates carrier emission from shallow traps (e.g., oxygen vacancies) that can significantly impact the performance of electronic devices.

#### 3.4.2. HIP-Induced Mechanism Transition

(1) Before HIP (Conventional Sintering):
  A total of 70% of GBs exhibit Ohmic conduction due to:
  Pore-induced conductive percolation (Figure 3a);
  Incomplete Bi diffusion (depth = 10 nm, Figure 3c);
  High oxygen vacancy density (1.2 × 10^21^ cm^−3^).
(2) After HIP (1100 °C/1.5 MPa):
  A total of 85% of GBs follow Schottky emission because:
  Densification eliminates pores (porosity < 0.05%, Figure 2b);
  Uniform Bi segregation forms a thick IBL (58 nm, Figure 4c);
  Oxygen vacancy density reduces to 2.1 × 10^21^ cm^−3^.

#### 3.4.3. Mechanistic Discussion

The dominant Schottky emission in HIP-treated GBs arises from:

(1) Barrier Height Enhancement: Uniform Bi segregation increases $\varphi_{B}$ by 77%, suppressing thermionic emission.
(2) Defect Suppression: Reduced oxygen vacancies and dislocations minimize trap-assisted conduction.
(3) IBL Thickness Effect: The 58 nm Bi diffusion layer provides a wider potential well, increasing the energy barrier for carrier transport.

In contrast, conventional GBs suffer from defect-induced Ohmic conduction, highlighting HIP’s role in transforming conduction mechanisms via microstructural optimization.

## 4. Conclusions

This study systematically analyzed the dielectric properties and microstructure of SrTiO_3_ grain boundary layer ceramics, focusing on the effects of Hot Isostatic Pressing (HIP) treatment. The results demonstrate that atmospheric pressure heat treatment results in low dielectric properties (dielectric constant: 10,000; breakdown strength: 555 kV/m), limiting the material’s potential for high dielectric applications due to poor oxidant penetration and the presence of numerous pores.

In contrast, optimizing the HIP process to 1100 °C and 1.5 MPa significantly improved the dielectric properties, yielding a high dielectric constant of 60,350 and a breakdown strength of 1722 kV/m. The HIP treatment facilitated uniform heating and high-pressure conditions that enhanced grain boundary reconstruction, atomic diffusion, and the penetration of Bi elements, leading to improved material densification and the elimination of pores.

These findings indicate that HIP treatment effectively enhances both the dielectric and insulating properties of SrTiO_3_ ceramics by optimizing the grain boundary structure. In our study, the dielectric material is intended for use in single-layer ceramic capacitors (SLCC), commonly used in various electronic applications. For comparison, current commercial dielectric materials produced by industry leaders such as Knowles, DLI, Tecdia, and Murata typically exhibit dielectric constants in the range of 50,000 to 60,000. However, these materials are designed for lower operating voltages, generally between 10 V and 16 V, with breakdown voltages around 100 V. In contrast, the dielectric material presented in our study exhibits superior dielectric and breakdown strength properties, making it suitable for more demanding applications.

Future work will explore the effect of HIP on multilayer ceramic capacitors, utilizing in situ TEM and phase-field modeling to further understand the relationship between grain boundary engineering and device reliability.

## Figures and Tables

**Table 1:** Thermal isostatic pressing process with SrTiO_3_ ceramics.

Sample	HIP Temperature (°C)	HIP Pressure (MPa)
1	Non-HIP	Non-HIP
2	1100	1.0
3	1100	1.5
4	1100	2.0
5	1000	1.5
6	1200	1.5

**Table 2:** Comparison of K value and loss of SrTiO_3_ grain boundary layer ceramics.

HIP Temperature (°C)	HIP Pressure (Mpa)	K	D (×10^−4^)
Non-HIP	Non-HIP	11,385	46
1000	1.5	31,280	33
1100	1.5	60,350	28
1100	2	57,880	43
1100	1	38,110	30
1200	1.5	55,180	32

Note: The data presented above are the average values derived from the test results of 20 samples for each type, with the standard deviation for each sample not exceeding 3%.

**Table 3:** Comparison of insulation resistance and breakdown voltage of SrTiO_3_ grain boundary layer ceramics.

HIP Temperature (°C)	HIP Pressure (MPa)	I.R (GΩ)	Breakdown Voltage (V)
Non-HIP	Non-HIP	0.8	110
1000	1.5	14	330
1100	1.5	13	310
1100	2	5	150
1100	1	3	120
1200	1.5	1.1	220

Note: The data presented above are the average values derived from the test results of 20 samples for each type, with the standard deviation for each sample not exceeding 3%.

## Data Availability

The original contributions presented in this study are included in the article. Further inquiries can be directed to the corresponding author.

## References

[1] Guo X., Pu Y.P., Ji J.M., Wang W., Li J.W., Shi R.K., Yang M. (2020). Colossal permittivity and high insulation resistivity in Dy-modified SrTiO_3_ lead-free ceramic materials with low dielectric loss. Ceram. Int..

[2] Ding H.W., Qiao Y.L., Wang M.Y., Li J., Tan X.Y. (2024). Synthesis and characterization of (sm, Al) co-doped SrTiO_3_ with giant dielectric constant and low dielectric loss through defect engineering design. J. Mater. Sci. Mater. Electron..

[3] Chen G., Chen J., Fu C., Peng X., Cai W., Deng X. (2015). Effect of Strontium Doping on the Microstructures and Dielectric Properties of Lanthanum Titanate Ceramics. Trans. Indian Ceram. Soc..

[4] Zhang X., Pu Y., Zhang L., Zhang J., Ning Y., Shang Y., Lu X., Zhang X. (2022). Simultaneously Achieving Colossal Permittivity, Ultralow Dielectric Loss Tangent, and High Insulation Resistivity in Er-Doped SrTiO_3_ Ceramics via Oxygen Vacancy Regulation. ACS Appl. Mater. Interfaces.

[5] Cordero F., Trequattrini F., Quiroga D., Silva P. (2021). Hopping and clustering of oxygen vacancies in BaTiO_3_ and the influence of the off-centred Ti atoms. J. Alloys Compd..

[6] Lin Y., Dylla M.T., Kuo J.J., Male J.P., Snyder G.J. (2020). Graphene/Strontium Titanate, Approaching Single Crystal-Like Charge Transport in Polycrystalline Oxide Perovskite Nanocomposites through Grain Boundary Engineering. Adv. Funct. Mater..

[7] Sohel M.P.M., Al Moyeen A., Safi A.I., Dipto M.S.R., Bin Syed S. (2025). Study of the structural, morphological, and J-V characteristics of Zr-doped barium calcium strontium titanate. J. Cryst. Growth.

[8] Lv Z.J., Lu M., Xiao G.Q., Ma Y., Weng D., Wang B., Shang Z., Qi W., Chen L., Chang J. (2025). A review of near-net shape forming by hot isostatic pressure technology. J. Am. Ceram. Soc..

[9] Maiwa H. (2014). Dielectric and Electromechanical Properties of BaTiO_3_ Ceramics Prepared by Hot Isostatic Pressing. Ferroelectrics.

[10] Kashif Hashmi M., Habib M.S., Waqar M., Hussain M.A., Iqbal A., Abbasi M., Saeed S. (2023). Synthesis and sintering of SrTiO_3_-ZnO ceramics, Role of ZnO content on microstructure and dielectric properties. Synth. Sinter..

[11] Tkach A., Okhay O., Almeida A. (2017). Giant dielectric permittivity and high tunability in Y-doped SrTiO_3_ ceramics tailored by sintering atmosphere. Acta Mater..

[12] Duan P., Liu P., Xu X., Wang W., Wan Z., Zhang S., Wang Y., Zhang J. (2017). Fabrication of transparent Tb_3_Al_5_O_12_ ceramics by hot isostatic pressing sintering. Compendex.

[13] Zhu D., Qian K., Pan Y., Hu Z., Li J. (2021). Fabrication and characterization of Ce,Y,SrHfO_3_ scintillation ceramics by hot isostatic pressing. J. Inorg. Mater..

[14] Kobune M., Nishioka Y., Yazawa T., Fujisawa H., Shimizu M. (2004). Ferroelectric properties and memory characteristics of Pb(Zr_0.52_Ti_0.48_)O_3_ thin films crystallized by hot isostatic pressing. Integr. Ferroelectr..

[15] Liu Q., Jing Y.Q., Su S., Li X.Y., Liu X., Feng Y.G., Chen X., Li J. (2020). Microstructure and properties of MgAl_2_O_4_ transparent ceramics fabricated by hot isostatic pressing. Opt. Mater..

[16] Zhou C., Liu X. (2008). Dielectric and piezoelectric properties of bismuth-containing complex perovskite solid solution of Bi_1/2_Na_1/2_TiO_3_–Bi(Mg_2/3_Nb_1/3_)O_3_. J. Mater. Sci..

[17] Li Z., Zhang D., Wang C., Zhang J., Wang Z., Wang Z., Yan X., Ai T., Wang D., Lu Z. (2023). The Influence of BaTiO_3_ Content on the Energy Storage Properties of Bi_0.5_Na_0.5_TiO_3_-Bi(Mg_2/3_Nb_1/3_)O3 Lead-Free Ceramics. Crystals.

[18] Jung W.K., Ko S.Y., Shin D., An S., Park J., Choi J., Lee K. (2024). Tailoring the microstructure of Ca-containing MgAl_2_O_4_ transparent ceramics prepared by sinter-HIP process. Ceram. Int..

[19] Rastogi B.K. (2005). Mesoscopic Simulation of Grain Boundary Diffusion Creep in Inhomogeneous Microstructures. Master’s Thesis.

[20] Dehghani P., Afghahi S., Soleimani F. (2025). Hot Isostatic Pressing (HIP) in Advanced Ceramics Production. Mater. Sci..

[21] Liu Y.A., Zhu J.Q., Dai B. (2020). Transparent MgAl2O4 ceramics prepared by microwave sintering and hot isostatic pressing. Ceram. Int..

[22] Arya B.B., Samantray N.P., Choudhary R.N.P. (2023). Sr(Sn,Se)O_3_ modified Bi_0.5_K_0.5_TiO_3_ ferroelectric ceramics, structural, electrical and leakage current characteristics. Appl. Phys. A Mater. Sci. Process..

[23] Sun F., Zhao H.F., Miao K. (2024). Influence mechanism of Ni_2_O_3_ doping on leakage current of SnO_2_ varistor ceramics. Int. J. Appl. Ceram. Technol..

[24] Premwichit P., Boonpang K., Chia J.Y., Nuntawong N., Prasertpalichat S. (2025). Novel complex-perovskite NaNbO_3_-Bi(Li_1/3_Hf_2/3_)O_3_ lead-free ceramics with improved energy-storage performance and reduced leakage current. J. Korean Ceram. Soc..

